# Low back pain research priorities: a survey of primary care practitioners

**DOI:** 10.1186/1471-2296-8-40

**Published:** 2007-07-11

**Authors:** Nicholas Henschke, Christopher G Maher, Kathryn M Refshauge, Anurina Das, James H McAuley

**Affiliations:** 1 Back Pain Research Group, School of Physiotherapy, University of Sydney, PO Box 170, Lidcombe, NSW 1825, Australia

## Abstract

**Background:**

Despite the large amount of time and money which has been devoted to low back pain research, successful management remains an elusive goal and low back pain continues to place a large burden on the primary care setting. One reason for this may be that the priorities for research are often developed by researchers and funding bodies, with little consideration of the needs of primary care practitioners. This study aimed to determine the research priorities of primary care practitioners who manage low back pain on a day-to-day basis.

**Methods:**

A modified-Delphi survey of primary care practitioners was conducted, consisting of three rounds of questionnaires. In the first round, 70 practitioners who treat low back pain were each asked to provide up to five questions which they would like answered with respect to low back pain in primary care. The results were collated into a second round questionnaire consisting of 39 priorities, which were rated for importance by each practitioner on a likert-scale. The third round consisted of asking the practitioners to rank the top ten priorities in order of importance.

**Results:**

Response rates for the modified-Delphi remained above 70% throughout the three rounds. The ten highest ranked priorities included the identification of sub-groups of patients that respond optimally to different treatments, evaluation of different exercise approaches in the management of low back pain, self-management of low back pain, and comparison of different treatment approaches by primary care professions treating low back pain.

**Conclusion:**

Practitioners identified a need for more information on a variety of topics, including diagnosis, the effectiveness of treatments, and identification of patient characteristics which affect treatment and recovery.

## Background

Low back pain imposes an enormous social and economic burden on society, and can be seriously disabling. International guidelines for the management of low back pain generally agree that the condition should principally be managed in primary care [1]. Back complaints represent a large proportion of the work of general practitioners, being one of the most common conditions for which patients seek care [2]. The question of how to successfully manage low back pain in primary care, however, remains unanswered.

The burden of low back pain on society continues to rise despite the vast amount of research and time devoted to its resolution. Many aspects of low back pain remain poorly understood, including risk factors for developing acute low back pain and attributing the cause of low back pain to a specific pathology [3]. Despite the large variety of treatments which have been evaluated through randomized controlled trials and meta-analyses, the effect sizes are often small, even for commonly used treatments such as exercise for chronic low back pain [4].

One potential explanation for the continued poor outcomes of low back pain, despite the attention it has received, is that research priorities are typically developed by government and industry authorities, funding agencies, and researchers [5,6] who can have agendas that differ from those of clinicians and patients. While these bodies may include clinicians in the agenda building process, the research being performed may not address the priorities of primary care practitioners who manage low back pain on a day-to-day basis. The clinical questions of most importance to primary care practitioners may not yet have been thoroughly investigated. In order to determine a research agenda that is relevant to primary care, we conducted a survey using a modified Delphi technique. We aimed to identify the low back pain research priorities of Australian primary care practitioners who manage the condition, and see whether these correspond to research priorities stated in Australian national clinical guidelines.

## Methods

### Study population

In Australia, the community primary care setting consists of a number of health care professions who provide health services to patients without the need for a prior referral. Low back pain is managed in primary care by three main professions in Australia, namely general practitioners, physiotherapists, and chiropractors. We identified practitioners in these three professions from clinics recruiting patients to a cohort study evaluating the prognosis of acute low back pain. All practitioners involved in the prognosis study were invited to participate. The study region was within the Sydney metropolitan area and was specifically chosen to cover a range of socio-economic values. Practitioners were provided with an information sheet regarding the modified-Delphi study and the first round questionnaire. If they consented to participate, they were asked to complete and return the questionnaire. All practitioners were experienced in managing patients with acute and chronic low back pain, and demonstrated continuing professional interest in improving the management of low back pain.

### The modified delphi technique

The Delphi technique is best-known as a survey method used to gain consensus among a group of respondents [7,8]. There are three main elements that characterise the Delphi technique and distinguish it from other group decision-making processes [7,8]. Firstly, distribution of the survey by mail guarantees anonymity of respondents to ensure that the influence of peer pressure on respondents' opinions might be minimised [7]. Another key element of the Delphi technique is that there is feedback by using successive questionnaires. Participants have the opportunity to reconsider their views, based on a summary of the group views. Finally, the Delphi technique uses a panel of 'experienced' or informed individuals.

There were three rounds to this survey with a two week interval between each of the three rounds. In round 1, participating practitioners were asked to list up to five of the most important questions about the management of low back pain in primary care that if addressed by researchers would improve their management of low back pain. Two authors collated the results and similar responses were collapsed to enable the development of the round 2 questionnaire. The intention was to maintain the subtlety and specific nature of as many of the responses as possible. Any discrepancies were resolved via consensus. In round 2 the participants were asked to rate the importance of the collated research priorities on a Likert scale from 0 to 5, with 0 meaning not at all important, and 5 meaning very important. In this way, practitioners are able to reconsider their responses while receiving feedback as to the responses of the rest of the practitioners. Round 3 consisted of the ten most important priorities, determined by median importance ratings, being returned to the practitioners in order. Where the median importance of the priorities was the same, the mode and then the mean was used to identify the top ten priorities. Each practitioner then ranked the ten priorities from 1 (highest) to 10 (lowest). In the second and third rounds of the Delphi, participants were asked to rate the importance of the priorities in general, not just the importance of those relevant to their practice.

### Analysis

The responses for round 3 were analysed using descriptive statistics, and ordered by the median rank. Where the median was equal between two priorities, the mode score was used to determine the higher rank. Inter-quartile ranges were inspected to determine the consistency of results within the sample. All statistical analyses were performed using SPSS v.12.0.1 (Chicago, ILL).

## Results

Eighty-five primary care practitioners were invited to participate, of which 70 (8 chiropractors, 16 general practitioners, and 46 physiotherapists) agreed to participate and completed the first round questionnaire. The practitioners were mostly male (56%) and had a mean (SD) of 14.8 (± 9.5) years of clinical experience. In total, 141 research priorities were identified by the 70 practitioners.

The 141 research priorities were collapsed to 39 discrete research priority topics for the second round questionnaire. The response rate for the second round was 74.2% (6 chiropractors, 14 general practitioners, and 32 physiotherapists). The mean importance scores were calculated for each topic, and the top ten priorities are presented in Table 1.

**Table 1:** Top ten low back pain (LBP) research priorities identified by primary care practitioners, and results from the round 3 ranking process.

**Final rank**	**Research priority (Which areas of low back pain management would you like more information on in order to improve your practice?)**	Median (IQR)*	**Mode**	**Rank after round 2**	**Present in Guidelines**
1	Identifying sub-groups of patients that respond optimally to different treatments	3.5 (1.75–7.00)	1	3	No
2	Core strengthening exercises as a treatment for LBP	4 (2.00–5.25)	1	2	Yes
3	Different exercise management approaches as a treatment for LBP	4 (2.00–6.00)	2	1	Yes
4	Self-management as a treatment of LBP	5 (3.00–7.00)	3	6	No
5	Manual therapy (mobilisation/manipulation) as a treatment for LBP	5 (3.75–8.00)	4	5	Yes
6	Factors that influence the process of recovery/recurrence	5 (3.00–6.00)	5	4	Yes
7	The importance of primary care in the treatment of LBP (when, who, how long)	6 (4.00–8.25)	9	9	No
8	Identifying a cause for LBP	7 (3.00–8.25)	8	8	No
9	Identification and treatment of patients with psychosocial "yellow flags"	8 (5.00–9.00)	9	10	Yes
10	Comparison of treatment approaches by primary care professions treating LBP	9 (5.00–10.00)	10	7	No

*IQR = inter-quartile range

These ten priorities were then sent back to the practitioners for the final ranking process. The response rate for round three was 71.4% (5 chiropractors, 14 general practitioners, and 31 physiotherapists). The median and mode rank after the third round can be seen in Table 1 for each priority. The first three priorities, based upon importance rating after round 2 remained the top three priorities after round 3. The inter-quartile ranges of the ranks show the diversity of opinion amongst the practitioners regarding the importance of each of the ten priorities. Following round 3, "Identifying sub-groups of patients that respond optimally to different treatments" became the highest ranked priority based on median rank. However, the large inter-quartile range (1.75–7.00) demonstrates a range of opinions among the respondents. Evaluation of core strengthening and other exercise approaches for the management of low back pain were the next two priorities.

Table 1 also illustrates whether a priority identified in this study was noted as a research priority in the current Australian guidelines for the management of acute low back pain [9]. Interestingly, half of the top ten priorities the practitioners identified coincided with the priorities in the guidelines. From the other perspective only 4 of the 14 priorities from the guidelines coincided with those that we identified (Table 2).

**Table 2:** Comparison of research priorities from Australian guidelines to those of primary care practitioners.

**Research Agenda from the NH&MRC Acute Low Back Pain Guidelines:**	**Primary care priority number**
Optimising the uptake of evidence-based guidelines by clinicians and consumers.	
International standardisation of definitions of intervention strategies and consistent outcome measures	
**Intervention studies addressing clinical and psychosocial predictors**	**9**
**Further research into secondary prevention of low back pain**	**6**
Evaluation of temperature treatments, ice, heat	
Evaluation of topical NSAIDs	
Evaluation of cox-2 NSAIDs, traditional NSAIDs, paracetamol and opioid analgesics	
**Evaluation of McKenzie therapy and other specific physical regimens**	**2, 3**
Evaluation of multi-disciplinary treatment (e.g. non-occupational settings, programmatic approaches to delivering multidisciplinary care)	
Evaluation of counseling and cognitive behavioural therapy	
**Evaluation of spinal manipulation (with and without prior x-ray)**	**5**
Evaluation of massage	
Evaluation of TENS in patients not responding to early advice to resume normal activities	
Evaluation of optimum combinations of therapies	

## Discussion

This study is the first to assess the low back pain research priorities of primary care practitioners. The top ten priorities identified in this study demonstrate that the primary care practitioners view low back pain within its broad clinical and social context, and suggest that research should be aimed towards developing more effective clinical tools (to classify patients into sub-groups, to identify psychosocial factors, and to treat successfully), and changing knowledge and behaviour of patients (self management and the importance of primary care). For primary care practitioners to successfully manage low back pain and for progress to be made in reducing the burden of the condition, they need answers to these questions.

Generally, clinical guidelines for the management of acute low back pain around the world provide similar recommendations for diagnosis and treatment [1]. Many also suggest that optimising the uptake of evidence-based guidelines by clinicians and consumers should be a major focus of future research [3,9]. In order for this to occur, however, research findings need to be relevant to the practitioners and other consumers. As this is the first study to develop research priorities exclusively from primary care practitioners, the priorities could only be compared to research agendas of published guidelines at a national level. Further studies in other countries are needed to determine whether the similarities and differences found by this comparison occur elsewhere around the world.

A number of similarities can be observed when a comparison is made between the research agenda in the Australian guidelines and the research priorities identified in this study by the primary care practitioners (Table 2). The identification of clinical and psychosocial predictors of chronicity, research into the secondary prevention of low back pain, the evaluation of specific physical regimens, and the evaluation of spinal manipulation were nominated as priorities for future research. Some of the priorities in the guidelines which were not identified by the practitioners were not directly relevant to management in primary care, such as the evaluation of multi-disciplinary treatment. Others were for further evaluation of seemingly common interventions such as massage and topical non-steroidal anti-inflammatory drugs (NSAIDs), the effectiveness of which are as yet unclear.

Five out of the top ten priorities identified by the practitioners in this study were not represented in the research agenda of the current Australian guidelines. In our view, the inclusion of these five priorities in research agendas is important to further the understanding and management of low back pain. By developing effective strategies for patients to self-manage their low back pain (priority 4) and gain further understanding of the role of the primary care setting (priority 7) and the professions involved (priority 10), the burden of low back pain on practitioners may be reduced. The presence of these issues within the top ten priorities of the practitioners, but not in the research agenda of the guideline seems anomalous given that guidelines generally recommend that low back pain should be managed in primary care [1].

Previous authors [5,6] have suggested that the identification of sub-groups of patients (priority 1) be the main priority of future back pain research. Whilst this was also the highest ranked priority of the primary care practitioners in this study, it is not present within the current Australian guideline. The classification of patients into sub-groups based on clinical, psychological, social, or other factors and determining whether they differ in response to treatments is important to ascertain which treatments will be most effective for which patients. Similarly, another priority that was identified by primary care practitioners but was not present in the research agenda of the Australian guidelines was that of diagnosing the cause of low back pain (priority 8). This is an important factor for practitioners and researchers alike, in order to further the understanding of the disease, and provide accurate information to patients regarding their condition and increase effectiveness of treatments. Despite acknowledging that 85% of low back pain cannot be attributed to a specific pathology, the guideline [9] does not recommend further research to determine the cause of the problem.

The choice of primary care practitioners for this study was based the need for an informed study population who would be able to provide research priorities using their knowledge of current low back pain research. Different priorities may have been raised by other primary care practitioners; however it was thought that any differences between them and the guidelines may simply reflect a lack of awareness of current research. Nevertheless, some of the priorities identified in this study have received a lot of research attention in the past, such as identifying a cause for low back pain. The inclusion of this priority, among others, may be influenced by the lack of consensus among the research community and the demands of patients in the primary care setting.

## Conclusion

Our results suggest that there are a number of similarities between the research questions that practitioners need answered and those promoted in clinical practice guidelines, but there are also important differences. Primary care practitioners identified a range of topics in need of further information including diagnosis, reducing the burden on primary care, and effectiveness of treatments. This study provides important information to researchers, and highlights the importance of including primary care practitioners in the development of a research agenda.

## Authors' contributions

NH, CGM, KMR, and JHM contributed to the design of the study. NH, AD, and JHM collected data and contributed to the analysis of the study. NH drafted the manuscript with input from the other authors. All authors read and approved the final manuscript.

## Pre-publication history

The pre-publication history for this paper can be accessed here:


